# Supplementary material for: Examining brain white matter after pediatric mild traumatic brain injury using neurite orientation dispersion and density imaging: An A-CAP study
Source: Neuroimage Clin. 2021 Nov 19;32:102887. doi: 10.1016/j.nicl.2021.102887 (PMC8633364; doi:10.1016/j.nicl.2021.102887)
Supplement: Supplementary data 1 [file mmc1.docx]

**Supplementary Table S1**. Table depicting means and standard deviations of DTI (FA, MD, AD, RD) and NODDI (NDI, ODI, FISO) metrics for each tract* and each hemisphere (left, right) for both groups (mTBI or OI) examined in this study.

| **Brain Tract** | **DTI/NODDI metric** | **mTBI** | | | | **OI** | | | |
| --- | --- | --- | --- | --- | --- | --- | --- | --- | --- |
|  |  | **Left** | | **Right** | | **Left** | | **Right** | |
|  |  | **Mean** | **Standard deviation** | **Mean** | **Standard deviation** | **Mean** | **Standard deviation** | **Mean** | **Standard deviation** |
| **AF** | | | | | | | | | |
|  | FA | .46 | .03 | .44 | .03 | .46 | .03 | .04 | .04 |
|  | MD (mm^2^/s) | .77x10^-3^ | .03x10^-4^ | .77x10^-3^ | .03x10^-4^ | .77x10^-3^ | .28x10^-4^ | .77x10^-3^ | .03x10^-4^ |
|  | AD | .12 | .02 | .12 | .02 | .12 | .02 | .12 | .02 |
|  | RD | .57 | .04 | .58 | .05 | .57 | .04 | .58 | .05 |
|  | NDI | .56 | .04 | .56 | .05 | .56 | .04 | .57 | .05 |
|  | ODI | .27 | .03 | .27 | .07 | .27 | .03 | .27 | .04 |
|  | FISO | .07 | .03 | .08 | .03 | .08 | .03 | .08 | .03 |
| **Cingulum** | | | | | | | | | |
|  | FA | .43 | .03 | .45 | .03 | .43 | .03 | .45 | .03 |
|  | MD (mm^2^/s) | .80x10^-3^ | .29x10^-4^ | .80x10^-3^ | .05x10^-4^ | .80x10^-3^ | .27x10^-4^ | .80x10^-3^ | .65x10^-4^ |
|  | AD | .12 | .02 | .12 | .02 | .12 | .02 | .12 | .02 |
|  | RD | .60 | .04 | .58 | .04 | .06 | .04 | .58 | .04 |
|  | NDI | .54 | .05 | .54 | .04 | .54 | .06 | .54 | .05 |
|  | ODI | .29 | .04 | .27 | .03 | .29 | .03 | .28 | .03 |
|  | FISO | .08 | .04 | .08 | .04 | .08 | .05 | .08 | .04 |
| **IFOF** | | | | | | | | | |
|  | FA | .47 | .02 | .46 | .03 | .47 | .03 | .47 | .03 |
|  | MD (mm^2^/s) | .82x10^-3^ | .29x10^-4^ | .83x10^-3^ | .31x10^-4^ | .82x10^-3^ | .27x10^-4^ | .83x10^-3^ | .29x10^-4^ |
|  | AD | .13 | .02 | .13 | .02 | .13 | .02 | .13 | .02 |
|  | RD | .59 | .04 | .59 | .04 | .59 | .04 | .59 | .04 |
|  | NDI | .52 | .04 | .52 | .04 | .53 | .04 | .53 | .04 |
|  | ODI | .24 | .03 | .25 | .06 | .25 | .03 | .25 | .03 |
|  | FISO | .08 | .03 | .09 | .03 | .08 | .03 | .09 | .03 |
| **ILF** | | | | | | | | | |
|  | FA | .46 | .02 | .45 | .03 | .46 | .02 | .45 | .03 |
|  | MD (mm^2^/s) | .83x10^-3^ | .30x10^-4^ | .83x10^-3^ | .33x10^-4^ | .83x10^-3^ | .28x10^-4^ | .83 x 10^-3^ | .30x10^-4^ |
|  | AD | .13 | .02 | .13 | .02 | .13 | .02 | .13 | .02 |
|  | RD | .59 | .04 | .60 | .05 | .59 | .04 | .60 | .04 |
|  | NDI | .53 | .04 | .52 | .04 | .54 | .05 | .53 | .04 |
|  | ODI | .25 | .03 | .26 | .07 | .25 | .03 | .26 | .04 |
|  | FISO | .09 | .03 | .08 | .03 | .09 | .03 | .09 | .03 |
| **CST** | | | | | | | | | |
|  | FA | .51 | .03 | .53 | .03 | .51 | .03 | .53 | .03 |
|  | MD (mm^2^/s) | .79x10^-3^ | .44x10^-4^ | .78x10^-3^ | .44x10^-4^ | .78x10^-3^ | .43x10^-4^ | .78x10^-3^ | .43x10^-4^ |
|  | AD | .13 | .02 | .13 | .03 | .13 | .02 | .13 | .02 |
|  | RD | .53 | .06 | .52 | .06 | .53 | .06 | .52 | .06 |
|  | NDI | .59 | .03 | .60 | .03 | .60 | .03 | .59 | .03 |
|  | ODI | .23 | .06 | .22 | .05 | .23 | .02 | .22 | .02 |
|  | FISO | .10 | .04 | .09 | .04 | .09 | .04 | .09 | .03 |
| **UF** | | | | | | | | | |
|  | FA | .41 | .03 | .40 | .03 | .42 | .03 | .40 | .03 |
|  | MD (mm^2^/s) | .84x10^-3^ | .24x10^-4^ | .84x10^-3^ | .23x10^-4^ | .84x10^-3^ | .23x10^-4^ | .84x10^-3^ | .22x10^-4^ |
|  | AD | .12 | .02 | .12 | .02 | .12 | .02 | .12 | .02 |
|  | RD | .63 | .03 | .64 | .03 | .63 | .03 | .64 | .03 |
|  | NDI | .47 | .04 | .46 | .04 | .47 | .04 | .47 | .05 |
|  | ODI | .26 | .04 | .27 | .05 | .26 | .03 | .27 | .03 |
|  | FISO | .05 | .03 | .05 | .03 | .06 | .04 | .06 | .04 |

DTI = Diffusion tensor imaging, NODDI = Neurite orientation dispersion and density index, AF = Arcuate fasciculus, ILF = Inferior longitudinal fasciculus, IFOF = Inferior fronto-occipital fasciculus, CST = Corticospinal tract, FA = Fractional anisotropy, MD = Mean diffusivity, NDI = Neurite density index, ODI = Orientation dispersion index, FISO = Fraction of isotropic water, *DTI metrics for the corpus callosum were not calculated for each hemisphere and have not been included here.

**Supplementary Table S2.** Fixed-Effects ANOVA results for DTI metrics (AD and RD) of 6 tracts, for each hemisphere (left and right). Significant p-values after FDR correction are **bolded**, significant p-values that did not survive FDR correction are *italicized*

| ***Predictor*** | ***F*** | ***p-values*** | | ***q-values*** | | ***_partial_ η^2^*** | | ***_partial_ η^2^***  ***90% CI***  ***[LL, UL]*** | |
| --- | --- | --- | --- | --- | --- | --- | --- | --- | --- |
| **AD** | | | | | | | | | |
| **AF** | | | | | | | | | |
| **model:** mean_h ~ Injury * dpi_mri * age + sex + Site_MRI | | | | | | | | | |
| Injury | 0.03 | | .859 | | .954 | | .00 | [.00, .00] | |
| dpi_mri | 1.51 | | .219 | | .686 | | .00 | [.00, .02] | |
| **age** | **18.13** | | **<.001** | | **<.001** | | **.00** | **[.01, .07]** | |
| *sex* | *6.60* | | *.010* | | *.035* | | *.00* | *[.00, .04]* | |
| **Site_MRI** | **3590.40** | | **<.001** | | **<.001** | | **1.00** | **[.97, .98]** | |
| Injury x dpi_mri | 0.02 | | .875 | | .875 | | .00 | [.00, .00] | |
| Injury x age | 0.05 | | .824 | | 1.000 | | .00 | [.00, .01] | |
| dpi_mri x age | 2.49 | | .115 | | .805 | | .00 | [.00, .02] | |
| Injury x dpi_mri x age | 0.09 | | .763 | | 1.000 | | .00 | [.00, .01] | |
| **CC**  **model:** mean_h ~ Injury * dpi_mri * sex + age + Site_MRI | | | | | | | | | |
| Injury | 1.09 | | .296 | | .527 | | .00 | [.00, .02] | |
| dpi_mri | 0.00 | | .964 | | .964 | | .00 | [.00, .00] | |
| sex | 0.96 | | .328 | | .383 | | .00 | [.00, .01] | |
| **age** | **56.70** | | **<.001** | | **<.001** | | **.10** | **[.07, .16]** | |
| **Site_MRI** | **3240.47** | | **<.001** | | **<.001** | | **1.00** | **[.97, .97]** | |
| Injury x dpi_mri | 0.15 | | .702 | | .819 | | .00 | [.00, .01] | |
| Injury x sex | 0.19 | | .662 | | 1.000 | | .00 | [.00, .01] | |
| dpi_mri x sex | 5.11 | | .024 | | .168 | | .00 | [.00, .03] | |
| Injury x dpi_mri x sex | 0.70 | | .403 | | 1.000 | | .00 | [.00, .01] | |
| **Cingulum**  **model:** mean_h ~ Injury * dpi_mri * sex + age + Site_MRI | | | | | | | | | |
| Injury | 1.60 | | .207 | | .527 | | .00 | [.00, .02] | |
| dpi_mri | 0.74 | | .391 | | .686 | | .00 | [.00, .01] | |
| sex | 1.83 | | .177 | | .248 | | .00 | [.00, .02] | |
| age | 2.27 | | .133 | | .133 | | .00 | [.00, .02] | |
| **Site_MRI** | **2294.37** | | **<.001** | | **<.001** | | **.94** | **[.96, .97]** | |
| Injury x dpi_mri | 0.44 | | .507 | | .710 | | .00 | [.00, .01] | |
| Injury x sex | 1.21 | | .271 | | 1.000 | | .00 | [.00, .02] | |
| dpi_mri x sex | 0.00 | | .963 | | 1.000 | | .00 | [.00, .01] | |
| Injury x dpi_mri x sex | 0.18 | | .671 | | 1.000 | | .00 | [.00, .01] | |
| **CST**  **model:** mean_h ~ Injury * dpi_mri * sex + age + Site_MRI | | | | | | | | | |
| Injury | 0.13 | | .723 | | .954 | | .00 | [.00, .01] | |
| dpi_mri | 0.73 | | .392 | | .686 | | .00 | [.00, .01] | |
| sex | 2.44 | | .119 | | .208 | | .00 | [.00, .02] | |
| **age** | **29.81** | | **<.001** | | **<.001** | | **.01** | **[.03, .10]** | |
| **Site_MRI** | **5767.05** | | **<.001** | | **<.001** | | **1.00** | **[.98, .99]** | |
| Injury x dpi_mri | 0.77 | | .381 | | .710 | | .00 | [.00, .01] | |
| Injury x sex | 0.32 | | .572 | | 1.000 | | .00 | [.00, .01] | |
| dpi_mri x sex | 0.50 | | .481 | | 1.000 | | .00 | [.00, .01] | |
| Injury x dpi_mri x sex | 0.20 | | .658 | | 1.000 | | .00 | [.00, .01] | |
| **IFOF** | | | | | | | | | |
| **model:** mean_h ~ Injury * dpi_mri * sex + age + Site_MRI | | | | | | | | |  |
| Injury | 0.00 | | .954 | | .954 | | .00 | [.00, .01] | |
| dpi_mri | 2.33 | | .128 | | .686 | | .00 | [.00, .02] | |
| sex | 0.01 | | .940 | | .940 | | .00 | [.00, .00] | |
| **age** | **33.17** | | **<.001** | | **<.001** | | **.01** | **[.03, .10]** | |
| **Site_MRI** | **3884.48** | | **<.001** | | **<.001** | | **1.00** | **[.97, .98]** | |
| Injury x dpi_mri | 0.53 | | .466 | | .710 | | .00 | [.00, .01] | |
| Injury x sex | 0.17 | | .677 | | 1.000 | | .00 | [.00, .01] | |
| dpi_mri x sex | 1.51 | | .219 | | .766 | | .00 | [.00, .02] | |
| Injury x dpi_mri x sex | 0.00 | | .959 | | 1.000 | | .00 | [.00, 1.00] | |
| **ILF**  **model:** mean_h ~ Injury * dpi_mri * age + sex + Site_MRI | | | | | | | | | |
| Injury | 1.07 | | .301 | | .527 | | .00 | [.00, .01] | |
| dpi_mri | 0.16 | | .688 | | .963 | | .00 | [.00, .01] | |
| **age** | **15.99** | | **<.001** | | **<.001** | | **.01** | **[.01, .06]** | |
| *sex* | *7.40* | | *.007* | | *.035* | | *.00* | *[.00, .04]* | |
| **Site_MRI** | **4945.73** | | **.000** | | **.000** | | **1.00** | **[.98, .98]** | |
| Injury x dpi_mri | 0.53 | | .467 | | .710 | | .00 | [.00, .01] | |
| Injury x age | 1.20 | | .275 | | .962 | | .00 | [.00, .02] | |
| dpi_mri x age | 0.34 | | .561 | | 1.000 | | .00 | [.00, .01] | |
| Injury x dpi_mri x age | 0.78 | | .376 | | 1.000 | | .00 | [.00, .01] | |
| **Uncinate**  **model:** mean_h ~ Injury * dpi_mri * age + sex + Site_MRI | | | | | | | | | |
| Injury | 2.22 | | .137 | | .527 | | .00 | [.00, .02] | |
| dpi_mri | 0.03 | | .853 | | .964 | | .00 | [.00, .00] | |
| age | 3.88 | | .050 | | .058 | | .00 | [.00, .03] | |
| sex | 3.67 | | .056 | | .131 | | .00 | [.00, .03] | |
| **Site_MRI** | **4941.90** | | **<.001** | | **<.001** | | **1.00** | **[.98, .98]** | |
| Injury x dpi_mri | 1.50 | | .221 | | .710 | | .00 | [.00, .02] | |
| Injury x age | 2.61 | | .107 | | .749 | | .00 | [.00, .02] | |
| dpi_mri x age | 0.01 | | .905 | | 1.000 | | .00 | [.00, .00] | |
| Injury x dpi_mri x age | 1.81 | | .179 | | 1.000 | | .00 | [.00, .02] | |
| **RD** | | | | | | | | | |
| **AF**  **model:** mean_h ~ Injury * dpi_mri * age + sex + Site_MRI | | | | | | | | | |
| Injury | 0.23 | | .631 | | .810 | | .00 | [.00, .01] | |
| dpi_mri | 0.34 | | .558 | | .639 | | .00 | [.00, .01] | |
| **age** | **23.67** | | **<.001** | | **<.001** | | **.04** | **[.02, .09]** | |
| sex | 2.26 | | .133 | | .310 | | .00 | [.00, .02] | |
| **Site_MRI** | **158.36** | | **<.001** | | **<.001** | | **.64** | **[.59, .67]** | |
| Injury x dpi_mri | 0.18 | | .670 | | .868 | | .00 | [.00, .01] | |
| Injury x age | 0.12 | | .726 | | 1.000 | | .00 | [.00, .01] | |
| dpi_mri x age | 0.39 | | .530 | | .742 | | .00 | [.00, .01] | |
| Injury x dpi_mri x age | 0.32 | | .575 | | 1.000 | | .00 | [.00, .01] | |
| **CC**  **model:** mean_h ~ Injury * dpi_mri * sex + age + Site_MRI | | | | | | | | | |
| Injury | 0.15 | | .694 | | .810 | | .00 | [.00, .01] | |
| dpi_mri | 1.27 | | .261 | | .639 | | .00 | [.00, .02] | |
| sex | 1.66 | | .198 | | .346 | | .00 | [.00, .02] | |
| **age** | **63.57** | | **<.001** | | **<.001** | | **.13** | **[.08, .17]** | |
| **Site_MRI** | **232.84** | | **<.001** | | **<.001** | | **.72** | **[.68, .74]** | |
| Injury x dpi_mri | 0.08 | | .775 | | .868 | | .00 | [.00, .01] | |
| Injury x sex | 0.60 | | .439 | | 1.000 | | .00 | [.00, .01] | |
| dpi_mri x sex | 2.39 | | .123 | | .430 | | .00 | [.00, .02] | |
| Injury x dpi_mri x sex | 0.98 | | .323 | | 1.000 | | .00 | [.00, .01] | |
| **Cingulum**  **model:** mean_h ~ Injury * dpi_mri * age + sex + Site_MRI | | | | | | | | | |
| Injury | 0.91 | | .341 | | .810 | | .00 | [.00, .01] | |
| dpi_mri | 0.22 | | .639 | | .639 | | .00 | [.00, .01] | |
| **age** | **19.96** | | **<.001** | | **<.001** | | **.03** | **[.02, .08]** | |
| *sex* | *7.50* | | *.006* | | *.021* | | *.00* | *[.00, .04]* | |
| **Site_MRI** | **116.70** | | **<.001** | | **<.001** | | **.56** | **[.51, .60]** | |
| Injury x dpi_mri | 0.29 | | .593 | | .868 | | .00 | [.00, .01] | |
| Injury x age | 0.70 | | .403 | | 1.000 | | .00 | [.00, .01] | |
| dpi_mri x age | 0.43 | | .511 | | .742 | | .00 | [.00, .01] | |
| Injury x dpi_mri x age | 0.13 | | .718 | | 1.000 | | .00 | [.00, .01] | |
| **CST**  **model:** mean_h ~ Injury * dpi_mri * age + sex + Site_MRI | | | | | | | | | |
| Injury | 0.01 | | .941 | | .941 | | .00 | [.00, .00] | |
| dpi_mri | 0.44 | | .507 | | .639 | | .00 | [.00, .01] | |
| **age** | **21.52** | | **<.001** | | **<.001** | | **.05** | **[.02, .08]** | |
| sex | 0.11 | | .736 | | 0.859 | | .00 | [.00, .01] | |
| **Site_MRI** | **578.40** | | **<.001** | | **<.001** | | **.86** | **[.85, .88]** | |
| Injury x dpi_mri | 0.03 | | .868 | | .868 | | .00 | [.00, .00] | |
| Injury x age | 0.09 | | .766 | | 1.000 | | .00 | [.00, .01] | |
| dpi_mri x age | 0.72 | | .396 | | .742 | | .00 | [.00, .01] | |
| Injury x dpi_mri x age | 0.20 | | .652 | | 1.000 | | .00 | [.00, .01] | |
| **IFOF**  **model:** mean_h ~ Injury * dpi_mri * sex + age + Site_MRI | | | | | | | | | |
| Injury | 0.42 | | .516 | | .810 | | .00 | [.00, .01] | |
| dpi_mri | 0.99 | | .319 | | .639 | | .00 | [.00, .01] | |
| sex | 0.37 | | .544 | | .762 | | .00 | [.00, .01] | |
| **age** | **130.78** | | **<.001** | | **<.001** | | **.23** | **[.16, .27]** | |
| **Site_MRI** | **168.25** | | **<.001** | | **<.001** | | **.64** | **[.59, .67]** | |
| Injury x dpi_mri | 0.76 | | .382 | | .868 | | .00 | [.00, .01] | |
| Injury x sex | 0.32 | | .569 | | 1.000 | | .00 | [.00, .01] | |
| dpi_mri x sex | 3.92 | | .048 | | .336 | | .00 | [.00, .03] | |
| Injury x dpi_mri x sex | 0.30 | | .583 | | 1.000 | | .00 | [.00, .01] | |
| **ILF**  **model:** mean_h ~ Injury * dpi_mri * age + sex + Site_MRI | | | | | | | | | |
| Injury | 0.60 | | .441 | | .810 | | .00 | [.00, .01] | |
| dpi_mri | 0.66 | | .415 | | .639 | | .00 | [.00, .01] | |
| **age** | **25.26** | | **<.001** | | **<.001** | | **.04** | **[.02, .08]** | |
| *sex* | *9.65* | | *.002* | | *.014* | | *.00* | *[.00, .04]* | |
| **Site_MRI** | **222.68** | | **<.001** | | **<.001** | | **.70** | **[.66, .72]** | |
| Injury x dpi_mri | 0.13 | | .716 | | .868 | | .00 | [.00, .01] | |
| Injury x age | 0.46 | | .497 | | 1.000 | | .00 | [.00, .01] | |
| dpi_mri x age | 1.15 | | .285 | | .742 | | .00 | [.00, .02] | |
| Injury x dpi_mri x age | 0.04 | | .839 | | 1.000 | | .00 | [.00, .00] | |
| **Uncinate**  **model:** mean_h ~ Injury * dpi_mri * age + sex + Site_MRI | | | | | | | | | |
| Injury | 1.41 | | .236 | | .810 | | .00 | [.00, .02] | |
| dpi_mri | 0.49 | | .483 | | .639 | | .00 | [.00, .01] | |
| **age** | **10.50** | | **.001** | | **.001** | | **.04** | **[.01, .05]** | |
| sex | 0.00 | | .964 | | .964 | | .00 | [.00, 1.00] | |
| **Site_MRI** | **102.46** | | **<.001** | | **<.001** | | **.52** | **[.46, .55]** | |
| Injury x dpi_mri | 0.51 | | .475 | | .868 | | .00 | [.00, .01] | |
| Injury x age | 1.04 | | .308 | | 1.000 | | .00 | [.00, .01] | |
| dpi_mri x age | 0.90 | | .343 | | .742 | | .00 | [.00, .01] | |
| Injury x dpi_mri x age | 0.27 | | .604 | | 1.000 | | .00 | [.00, .01] | |

*Note.* LL and UL represent the lower-limit and upper-limit of the partial η^2^ confidence interval, respectively. q-value is the expected proportion of false positives incurred when calling a test significant using FDR correction.^51^ DTI = Diffusion tensor imaging, AD= Axial diffusivity, RD = Radial diffusivity, AF = Arcuate fasciculus, ILF = Inferior longitudinal fasciculus, IFOF = Inferior fronto-occipital fasciculus, CST = Corticospinal tract, UF = Uncinate fasciculus, CC = Corpus callosum.
